# Application of Nanodrug Delivery Systems in Enhancing Treatment of Gastritis and Gastric Cancer: A Systematic Evaluation of Targeted Therapy

**DOI:** 10.3390/pharmaceutics17060683

**Published:** 2025-05-22

**Authors:** Miaomiao Xu, Shujie Tian, Jing Wang, Shuqing Gan, Ziting Zhang, Lixing Weng

**Affiliations:** State Key Laboratory of Flexible Electronics (LoFE), Institute of Advanced Materials (IAM), Nanjing University of Posts and Telecommunications, Nanjing 210049, China; xumiaomiao@njupt.edu.cn (M.X.); 1023173007@njupt.edu.cn (S.T.); 1223066422@njupt.edu.cn (J.W.); 1024233702@njupt.edu.cn (S.G.); 1024233719@njupt.edu.cn (Z.Z.)

**Keywords:** nanomedicine, gastritis, gastric cancer, clinical translation, stability, precision therapy

## Abstract

In recent years, nanomedicine has been emerging as a promising therapeutic approach in the treatment of gastritis and gastric cancer, particularly through targeted drug delivery systems and combination therapies that enhance therapeutic effects. Gastritis and gastric cancer, being common gastrointestinal diseases, often exhibit suboptimal treatment outcomes due to the limitations of traditional medications. Interventions based on nanotechnology not only improve the local concentration and bioavailability of drugs but also promote precise targeted therapy by regulating drug release rates, while minimizing adverse side effects, thereby enhancing therapeutic efficacy. Despite significant progress in basic research and preclinical applications, the clinical translation of nanomedicine still faces numerous challenges, including stability, biocompatibility, production standardization, regulatory and ethical barriers, as well as optimization of clinical trial designs. Furthermore, combining nanomedicine with other therapeutic modalities, such as immunotherapy and gene therapy, may open new avenues for addressing complex digestive system diseases. Future research should continue to explore the potential of nanocarriers, particularly in the formulation and stability of nanomaterials for precision therapy, with the aim of improving the quality of life and survival rates for patients with gastritis and gastric cancer.

## 1. Introduction

Gastritis, a globally prevalent gastrointestinal disorder, imposes a substantial clinical burden, affecting the quality of life for hundreds of millions of individuals [1]. The pathogenesis of gastritis and its progression to gastric cancer is a complex biological process [2] involving multiple factors and mechanisms [3,4]. Compelling evidence establishes *Helicobacter pylori* (*H. pylori*) infection as a principal etiological agent in chronic gastritis and its associated sequelae, including peptic ulcer disease and gastric carcinogenesis [5]. The underlying pathogenesis involves intricate crosstalk between bacterial virulence determinants and host immune defense mechanisms [6,7]. While conventional therapeutic regimens combining antibiotics with proton pump inhibitors (PPIs) demonstrate efficacy in ameliorating *H. pylori*-induced pathology, their clinical utility is constrained by variable therapeutic responses, adverse effect profiles, and the escalating crisis of antimicrobial resistance [8]. These limitations underscore the imperative for novel therapeutic interventions.

Nanomedicine encompasses engineered therapeutic platforms operating at the nanoscale (1–100 nm), leveraging distinctive physicochemical properties to revolutionize drug delivery paradigms [9]. These systems offer transformative advantages including spatiotemporal control of drug release, enhanced pharmacokinetic profiles, and improved biocompatibility, thereby addressing critical limitations of conventional pharmacotherapy [10,11,12]. Recent breakthroughs in nanotherapeutic design have catalyzed innovative approaches to gastritis and gastric cancer management. Notably, advanced nanocarriers such as lipid-based and polymeric nanoparticles exhibit enhanced drug stability and prolonged systemic circulation while achieving targeted accumulation in diseased gastric mucosa [13,14]. Such platforms show particular promise in augmenting *H. pylori* eradication efficacy and therapeutic precision [15]. Nevertheless, the translational pathway for nanomedicine in gastroenterology confronts substantial barriers, including material biocompatibility, formulation stability, manufacturing scalability, and the imperative for patient-specific therapeutic solutions [16].

Notwithstanding these challenges, nanomedicine represents a paradigm-shifting frontier in combating *H. pylori* persistence [17]. Contemporary research efforts are focused on engineering multifunctional nanocarrier systems with optimized tropism for *H. pylori*-infected microenvironments, thereby potentiating therapeutic outcomes in gastropathology [6]. Against this backdrop, this article aims to provide a comprehensive overview of the latest advancements in nanomedicine, with a particular focus on formulation and targeted drug delivery strategies, and their therapeutic potential in the treatment of gastritis and gastric cancer. By exploring these developments, nanomedicine is expected to offer novel therapeutic approaches for improving clinical outcomes in patients with gastritis and gastric cancer, thereby contributing to the advancement of personalized medicine.

## 2. The Preparation, Targeted Delivery, and Advantage of Nanomedicine

### 2.1. The Preparation of Nanomedicine

**Figure 1:**
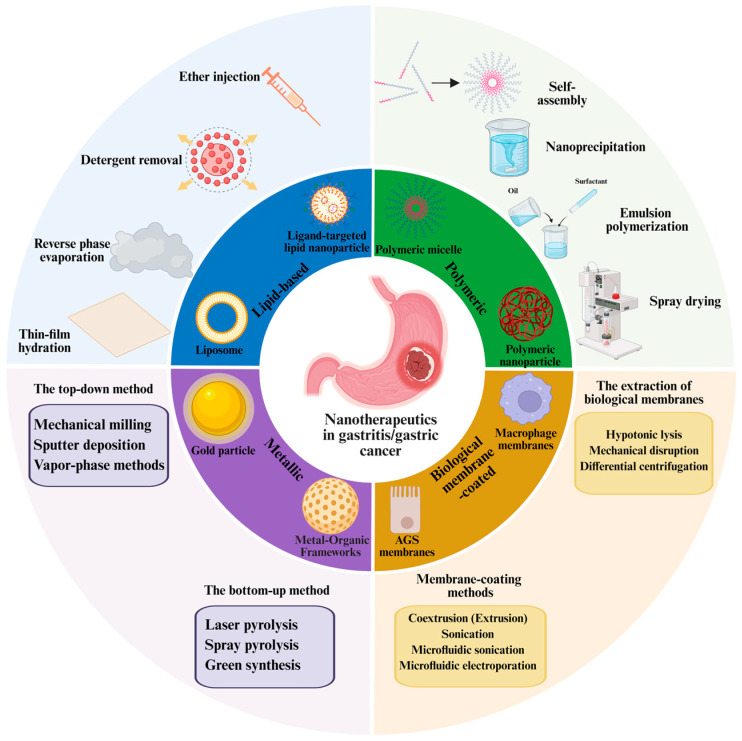
Four nanoparticle platforms and their preparation methods for gastritis and gastric cancer therapy: lipid-based nanoparticles, polymeric nanoparticles, metallic nanoparticles, and biological membrane-coated nanoparticles.

Nanomedicines can be systematically classified based on their composition and structural characteristics. The primary categories include the following: (1) lipid-based nanoparticles, (2) polymeric nanoparticles, (3) metallic nanoparticles, and (4) biological membrane-coated nanoparticles (Figure 1). The preparation of nanoparticles constitutes a fundamental aspect of nanomedicine, encompassing a variety of techniques designed to ensure efficient drug delivery. This section outlines several commonly employed preparation methods for nanomedicine (Figure 1).

(1) The preparation methods for lipid-based nanoparticles

Traditional approaches to lipid-based nanoparticles include thin-film hydration (TFH), reverse-phase evaporation (RPE), emulsification, detergent removal, and solvent injection. In the TFH method, lipid materials are typically dissolved in chloroform [18,19,20] or a mixture of chloroform and a polar solvent. The solvent is then evaporated to form a thin lipid film. The RPE method represents an enhanced version of TFH. Initially, lipids are dissolved in a water-immiscible organic solvent, followed by the addition of an aqueous solution to form a water-in-oil (W/O) emulsion. Subsequent evaporation of the organic solvent leads to the formation of a semi-solid gel, which is then dispersed into an aqueous phase to yield a liposome suspension [21,22]. The emulsification technique shares the initial steps with RPE. However, in the third step, the W/O emulsion is mixed with a large volume of aqueous solution to produce a water-in-oil-in-water (W/O/W) double emulsion. The removal of the organic solvent by evaporation subsequently results in liposome formation [23].

The detergent removal method is primarily employed for the preparation of unilamellar liposomes and is especially useful in membrane protein reconstitution. In this approach, phospholipid–detergent micelles are first formed. Detergents are then removed through dialysis, column chromatography, or adsorption onto biobeads [21], facilitating the self-assembly of liposomes.

In the solvent injection method, lipids are dissolved in a water-miscible organic solvent, most commonly ethanol [24,25]. The ethanolic lipid solution is then injected into an aqueous phase using either a manual syringe or an automated pump. During this process, the aqueous phase is preheated to a temperature above the lipid phase transition point. Once liposomes are formed, residual ethanol is removed under reduced pressure using a rotary evaporator.

(2) The preparation methods for polymeric nanoparticles

Self-assembly represents one of the most prevalent strategies for the fabrication of polymeric nanoparticles. This process is governed by a variety of supramolecular interactions, including hydrophobic interactions, hydrogen bonding, electrostatic forces, and van der Waals interactions. Among these, hydrophobic interactions are widely recognized as the primary driving force behind the self-assembly of polymeric nanoparticles [26].

Another widely adopted method for preparing polymeric nanoparticles is nanoprecipitation (also known as solvent displacement or desolvation). This technique, also based on polymer self-assembly, involves initially dissolving the polymer in a water-miscible organic solvent, which is then gradually introduced into an aqueous phase to induce nanoparticle formation [27]. Due to its operational simplicity and ease of implementation, nanoprecipitation has become one of the most widely utilized techniques in the pharmaceutical industry for nanoparticle synthesis. Recent advancements in microfluidic technologies have significantly addressed the limitations of traditional nanoprecipitation methods, particularly the issue of particle size heterogeneity [28].

In contrast to the rapid solvent exchange involved in nanoprecipitation, dialysis enables the gradual removal of organic solvent, thereby facilitating polymer self-assembly into nanoparticles over time [29]. Moreover, by modulating the pH of the dialysate, pH-responsive nanoparticles can be generated, offering additional functionality for targeted drug delivery applications [30].

Emulsion-based methods are also frequently employed in the fabrication of polymeric nanoparticles and include both emulsion self-assembly and emulsion polymerization [31]. In emulsion self-assembly, preformed polymers are dissolved in an organic solvent immiscible with water. Upon addition to an aqueous phase and application of vigorous stirring, homogenization, or ultrasonication, an oil-in-water (O/W) emulsion is formed. As the organic solvent is removed, the polymer self-assembles into nanoparticles.

In contrast, emulsion polymerization involves polymerizing monomers within O/W emulsions, leading to nanoparticle formation inside the emulsion droplets. This approach is advantageous for scalable and industrial-scale production due to its high reproducibility and process control.

Additionally, ionotropic gelation is a well-established technique for the preparation of polymeric nanoparticles. This method relies on the electrostatic interaction between polyelectrolytes and suitable counter-ions or polyelectrolyte complexes, resulting in the formation of nanoparticulate gels [32]. It is particularly suitable for the self-assembly of charged macromolecules. For instance, positively charged chitosan can interact with anionic phosphate polymers to form nanoparticles [33]. Due to their surface charge, these nanoparticles are widely utilized as carriers for nucleic acid-based bioactive compounds.

Beyond techniques based on physicochemical triggers, spray drying offers a mechanical approach to nanoparticle preparation by atomizing a fluid into fine powders. This method enables the production of nanoparticles with diameters below 200 nm and is particularly advantageous for the fabrication of nanoparticles intended for oral and inhalation drug delivery [34].

(3) The preparation methods for metallic nanoparticles

The synthesis of metallic nanoparticles can generally be categorized into two main approaches: the top-down method and the bottom-up method.

The top-down approach involves reducing bulk metal materials from the millimeter scale to the nanoscale using physical or chemical techniques such as mechanical milling, sputter deposition, and vapor-phase methods [35]. This strategy is capable of producing bimetallic, trimetallic, or even multimetallic nanoparticles, offering versatility in the composition of the resulting nanomaterials.

In contrast, the bottom-up approach, also known as the construction method, relies on the assembly of smaller building blocks such as atoms or molecules to form nanoparticles. Techniques such as laser pyrolysis, spray pyrolysis, and green synthesis fall under this category. Notably, metal–organic frameworks (MOFs) are predominantly synthesized using bottom-up strategies, allowing for precise control over structure and composition [36].

(4) The preparation methods for biomembrane-coated nanoparticles

The preparation of biomembrane-coated nanoparticles generally involves two key steps: the extraction of biological membranes and the fabrication of the nanoparticle core. Biological membranes can be isolated through techniques such as hypotonic lysis, mechanical disruption, and differential centrifugation.

The nanoparticle core may consist of either organic materials (such as poly (lactic-co-glycolic acid) (PLGA) or albumin) or inorganic materials (such as gold, silver, or iron oxide). Once both components are prepared, the biological membrane is coated onto the nanoparticle core surface using a variety of established techniques. Common membrane-coating methods include coextrusion (extrusion), sonication, microfluidic sonication, and microfluidic electroporation.

Coextrusion involves the repeated extrusion of a mixture of cell membrane vesicles and nanoparticles through a porous polycarbonate membrane, facilitating membrane fusion and nanoparticle encapsulation. This method yields uniform coatings with high reproducibility, though it is relatively time-consuming [37]. Sonication utilizes ultrasonic cavitation to transiently disrupt the membrane structure, thereby promoting rapid and efficient membrane coating of nanoparticles. This method also reduces material waste; however, it may result in non-uniform membrane coverage [38]. Microfluidic sonication combines ultrasonic energy within a microfluidic chip to rapidly complete the coating process. The coating efficiency can reach up to 93%, and continuous production can be achieved [37,39]. Microfluidic electroporation creates temporary pores in the membrane using electric pulses, allowing nanoparticles to enter the membrane. This enables high-precision and high-throughput coating, but the equipment is complex and expensive [40,41].

Apart from the preparation method, the formulation stability is a critical determinant in the clinical translation of nanomedicines. The in vivo stability of nanoparticles not only influences the drug release profile but also directly affects their therapeutic efficacy and safety. Extensive studies have demonstrated that the physicochemical properties of nanoparticles, such as particle size, surface charge, and morphology, play a pivotal role in determining their stability [42]. For example, nanoparticles with smaller diameters typically exhibit higher surface energy, which can promote particle aggregation and compromise formulation performance.

To address these challenges, significant efforts have been directed toward the optimization of formulation compositions and manufacturing processes to enhance the stability of nanoparticles. The incorporation of appropriate stabilizing agents, such as polyethylene glycol (PEG) and its derivatives or chitosan-based polymers, has been shown to effectively inhibit nanoparticle aggregation and prolong systemic circulation time [6].

Moreover, external environmental factors, including pH, temperature, and ionic strength, can further impact nanoparticle stability. Therefore, comprehensive stability evaluations are essential during the formulation development phase. These often include in vitro assessments under physiologically relevant conditions, such as pH- and temperature-dependent release studies, to ensure the safety, efficacy, and reliability of the final nanomedicine product in clinical applications [42].

Thus, in actual production, it is necessary to choose a suitable preparation method according to the requirements of the application, including biocompatibility, scalability, and so on.

### 2.2. The Targeted Delivery of Nanomedicine

Nanodelivery systems, as an emerging drug delivery technology, have demonstrated considerable potential in the treatment of gastritis and gastric cancer in recent years. By leveraging the distinct pathological characteristics of these conditions, nanotechnology-based approaches enable targeted drug delivery to lesion sites, thereby enhancing drug bioavailability and therapeutic efficacy while minimizing systemic adverse effects.

(1) Nanomedicine targeted to Gastritis

*H. pylori* infection is a primary etiological factor in gastritis and peptic ulcer disease. However, conventional antibiotic therapies for *H. pylori* eradication lack tissue specificity, leading to two critical complications: the emergence of antibiotic-resistant bacterial strains and disruption of intestinal microbiota homeostasis [43]. Consequently, the rational design of nanoparticles tailored to *H. pylori*’s unique biological characteristics for targeted delivery to infection sites represents a crucial advancement in precision therapy for gastritis.

**Figure 2:**
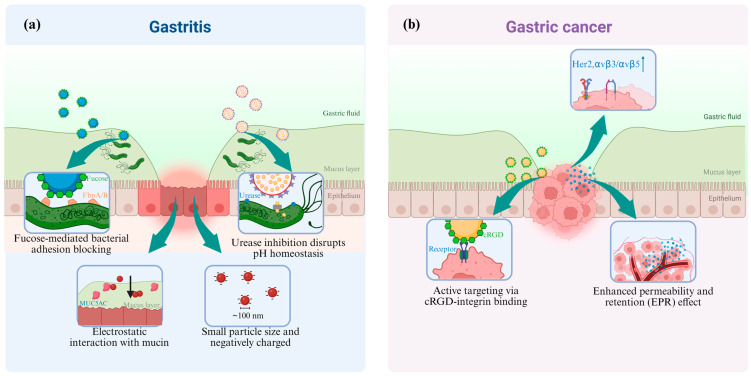
Action sites and mechanisms of nanoparticles in treating gastritis and gastric cancer.

Targeted drug delivery strategies for *H. pylori* eradication leverage its surface adhesion factors through dual mechanisms. First, nanoparticles functionalized with fucose-based ligands, such as HpaA adhesin-binding moieties, exploit the bacterium’s fucose-binding proteins to enhance drug enrichment on bacterial surfaces [44]. Second, urea-conjugated nanocarriers, such as chitosan nanoparticles, utilize the high-affinity interaction between urease channel proteins and urea substrates (Figure 2a). This dual approach achieves two therapeutic objectives: (1) direct antibiotic delivery into bacterial cytoplasm via urea transporter-mediated internalization and (2) suppression of urease activity to disrupt pH homeostasis, thereby sensitizing *H. pylori* to gastric acidity [45].

Mucoadhesive nanocarriers modified with bioadhesive materials (pectin, chitosan, or hyaluronic acid (HA)) demonstrate enhanced gastric retention through electrostatic interactions with mucin glycoproteins in the gastric mucosa, thereby prolonging therapeutic exposure at infection sites [46].

Furthermore, nanoparticles featuring small particle sizes (~100 nm) and negatively charged surfaces, such as fucoidan-modified systems, exhibit dual functionality: (1) efficient penetration through the gastric mucus barrier via optimized physicochemical interactions and (2) targeted disruption of *H. pylori* biofilms within deep mucosal layers. This synergistic mechanism enables precise eradication of bacterial colonies while preserving gastrointestinal microbiota homeostasis, as evidenced in advanced nanotherapeutic platforms employing ultrasound-activated radical generation [44].

(2) Nanomedicine targeted to Gastric Cancer

Gastric cancer remains a major global health burden, ranking as the fifth most prevalent malignancy and the fourth leading cause of cancer-related mortality worldwide. The disease is characterized by poor prognosis and suboptimal therapeutic outcomes with conventional chemotherapy. To address these challenges, nanotechnology-driven strategies for gastric cancer treatment focus on three principal approaches: active targeting, passive targeting, and pH-responsive drug release (Figure 2b).

Engineered nanoparticles featuring surface-modified cRGD peptides demonstrate active targeting capabilities toward tumor vasculature and gastric cancer tissues by selectively engaging overexpressed integrin receptors αvβ3/αvβ5. Enhanced specificity arises from ligand–receptor interactions, facilitating receptor-mediated endocytosis for cellular internalization. This targeted delivery paradigm achieves superior therapeutic drug accumulation at tumor sites [47]. Additionally, nanocarriers can be functionalized with receptor-specific binding moieties targeting alternative overexpressed receptors, such as human epidermal growth factor receptor 2 (HER2), epidermal growth factor receptor (EGFR), fibroblast growth factor receptor 2 (FGFR2), Mesenchymal–epithelial transition factor (MET), thereby enabling precise drug delivery through multimodal targeting mechanisms [47].

The passive targeting of nanoparticles utilizes the enhanced permeability and retention (EPR) effect in tumor tissues, enabling nanoparticles to more easily penetrate tumor vasculature and accumulate within the tumor microenvironment.

Gastric cancer tissues exhibit an acidic microenvironment (pH = 5.5–6.8), enabling pH-responsive nanoparticle systems to achieve tumor-specific drug release. For example, ZIF-90 nanoparticles demonstrate pH-dependent degradation kinetics, disintegrating rapidly in acidic tumor conditions to release paclitaxel with 89% efficiency within 24 h, whereas only <15% release occurs in physiological pH (7.4) or alkaline normal tissues (pH = 8.0). This tumor-selective release mechanism enhances therapeutic efficacy by minimizing systemic cytotoxicity while maximizing tumor site drug concentration [48].

### 2.3. The Advantage of Nanomedicine

Nanomedicines exhibit multiple superior characteristics in drug delivery applications. Primarily, through surface charge modulation or amination functionalization, nanocarriers demonstrate enhanced mucus-penetrating capacity, facilitating efficient tissue targeting and improved therapeutic outcomes [49]. Secondly, the nanoscale dimensions enable passive accumulation at tumor or inflammatory sites via the EPR effect, achieving localized drug enrichment while minimizing systemic toxicity [50]. Furthermore, surface functionalization with targeting moieties, such as antibodies, peptides, and small-molecule ligands, significantly improves cellular internalization efficiency, thereby enhancing treatment specificity and efficacy [51].

These nanoplatforms possess remarkable stimuli-responsive drug release capabilities, responding to either exogenous triggers (light, ultrasound, and magnetic fields) or endogenous microenvironmental cues (pH, ROS levels, and enzymes) [1]. For instance, near-infrared light can activate photothermal–chemotherapy hybrid systems to disrupt nanocarrier structures for controlled drug release [52], while pH-sensitive nanogels undergo acid-triggered degradation for pulsatile antibiotic delivery in infected microenvironments [53]. Advanced systems incorporating multiple responsive mechanisms, such as ROS and magnetic responsive, demonstrate synergistic effects against drug resistance [54]. Despite challenges posed by microenvironmental complexity, these intelligent nanosystems represent a revolutionary approach to gastritis therapy.

The pharmacological efficacy of nanomedicines in gastritis treatment stems from their unique physicochemical properties and biocompatibility advantages [55]. Compared to conventional formulations, nanocarrier systems substantially enhance gastrointestinal bioavailability and targeting efficiency, overcoming multiple absorption barriers in the complex gastrointestinal milieu [56]. Recent advances highlight surface engineering as a pivotal strategy, where precise modulation of surface charge distribution, hydrophilicity, and particle size distribution optimizes gastric mucosa-targeted delivery [57]. Moreover, controlled release kinetics prolong systemic circulation time, simultaneously improving therapeutic effects while reducing adverse reactions [58].

In combating *H. pylori*-associated gastritis, nanomedicines employ multiple bactericidal mechanisms: (1) mimicking host–pathogen interactions through adhesion blockade or channel targeting; (2) exploiting bacterial-specific components (such as urease channels and membrane receptors); and (3) utilizing physicochemical properties (such as pH responsiveness) to disrupt bacterial membranes or interfere with metabolic pathways [59]. Innovative designs combining antibiotics with anti-inflammatory agents in multifunctional nanoplatforms further demonstrate synergistic therapeutic effects [60], representing a paradigm shift in gastritis management.

## 3. Therapeutic Applications of Nanomedicine in Gastritis

Recent years have witnessed significant advancements in the application of nanomedicine for gastritis treatment. Numerous studies have demonstrated that nanocarriers can effectively enhance drug bioavailability and therapeutic efficacy.

### 3.1. Lipid-Based Nanoparticles

Lipid-based nanoparticles have emerged as promising therapeutic vehicles for gastritis treatment. The growing prevalence of antibiotic-resistant *H. pylori* infections, strongly associated with biofilm formation capacity, represents a critical challenge in clinical management. To address this, Professor Hu’s research group engineered an innovative liposomal–polymeric hybrid nanoplatform. The nanoparticles were composed of chitosan nanoparticles (CS NPs) as the core and a mixed lipid layer containing rhamnolipid (RHL) as the shell, and they were modified with DSPE-PEG2000 to enhance hydrophilicity. Clarithromycin (CLR), a first-line antibiotic, was encapsulated within the nanoparticles. The assembly process utilized a two-step method: CS N

**Figure 3:**
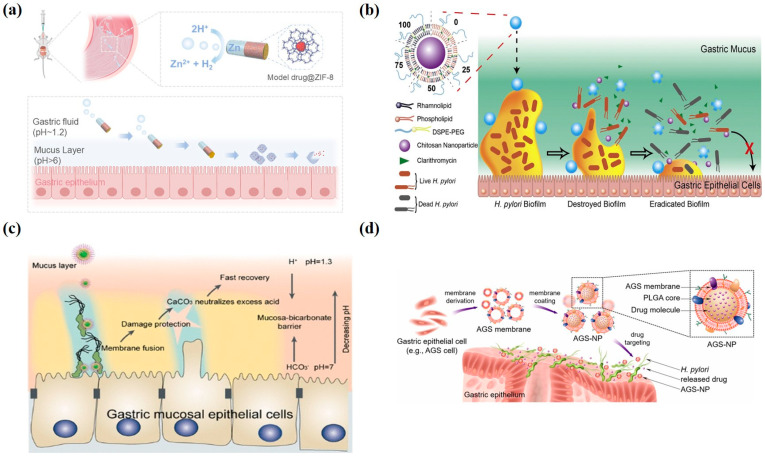
Therapeutic applications of nanomedicine in gastritis. (**a**) Schematic illustration of the self-propelled microrockets for controlled and sustained drug delivery [reprinted with permission from Ref. [36]; Copyright (2025) American Chemical Society]. (**b**) Structure of LPNs and schematic of the process by which LPNs eradicate bacterial biofilms [reprinted with permission from Ref. [61]; Copyright (2019) Elsevier]. (**c**) Antibacterial mechanism illustration of CaCO_3_@Fe–TP@EggPC nanoliposomes (CTE NLs) loaded with tea polyphenols (TPs) [reprinted with permission from Ref. [62]; Copyright (2022) American Chemical Society]. (**d**) Preparation of AGS nanoparticles and their schematic representation for targeted antibiotic delivery to *H. pylori*-infected sites [reprinted with permission from Ref. [66]; Copyright (2018) Wiley].

Ps were first prepared via ionotropic gelation, followed by solvent evaporation and high-pressure homogenization to encapsulate the lipid layer onto the CS NPs. The RHL content in the lipid layer ranged from 0% to 100% to evaluate its impact on the nanoparticles’ anti-biofilm activity. The nanoparticles exerted their mechanisms through three pathways: RHL disrupted the biofilm matrix within the lipid shell, CLR and CS NPs exerted antibacterial effects against biofilm bacteria, and both CS NPs and RHL inhibited bacterial adhesion and biofilm formation. Experimental results demonstrated that the lipid–polymer hybrid nanoparticles (LPNs) significantly reduced biofilm biomass, decreased biofilm viability, disrupted biofilm structure, and eliminated extracellular polymeric substances (EPSs) (Figure 3b) [61].

The therapeutic efficacy of gastritis treatments is frequently compromised by gastric acid interference. To address this challenge, pH-modulating nanoliposomes have emerged as a promising strategy. Professor Han’s research team developed CaCO_3_@Fe–TP@EggPC nanoliposomes (CTE NLs) with a unique ternary architecture: (1) a calcium carbonate (CaCO_3_) core for acid neutralization, (2) iron (III)-tea polyphenol (Fe^3+^-TP) coordination complexes on the core surface, and (3) an outer phosphatidylcholine bilayer. This design conferred three synergistic therapeutic functions: First, the CaCO_3_ core rapidly buffered excess gastric acid. Second, the Fe^3+^-TP coordination complexes selectively fused with *H. pylori* membranes, demonstrating potent bactericidal activity. Third, the tea polyphenols concurrently ameliorated systemic inflammation and modulated gut microbiota. Compared to the control group, CTE NLs elicited exceeding 80% attenuation in biofilm biomass and achieved a 90% reduction in gastric *H. pylori* burden in mice, thereby demonstrating marked anti-biofilm and bactericidal efficacy (Figure 3c) [62].

### 3.2. Polymeric Nanoparticles

*H. pylori* is a primary pathogen in chronic gastritis and gastric ulcers. Traditional antibiotic treatments are limited by rising microbial resistance and cytotoxicity. Bruna’s research team addressed these challenges by encapsulating a silver (I) coordination complex (compound **1**) within poly (ε-caprolactone) (PCL)/poloxamer 407 (PLX) nanoparticles. Compared to free compound **1** (MIC = 3.90 μg/mL), the nanosystem achieved a five-fold increase in bactericidal activity (MIC = 0.781 μg/mL) and suppressed biofilm formation by 85%. Under acidic gastric conditions (pH = 1.2), sustained drug release (>72 h) enhanced mucosal retention. In vitro assays revealed improved biocompatibility compared to the parent compound. These findings validate polymer–nanoparticle encapsulation as a promising strategy for targeted *H. pylori*-associated gastritis therapy [63].

### 3.3. Metallic Nanoparticles

Metal-based nanoparticles have attracted significant attention due to their unique physicochemical properties, including metal ion release, reactive oxygen species (ROS) generation, and photothermal/photodynamic effects [64]. Zhou developed a selenized chitosan-modified bismuth-based metal–organic framework (Bi-MOF@CS-Se) nanodrug. The CS-Se shell mediated mucosal adhesion and gastric retention via electrostatic interactions, while gastric acid/pepsin exposure selectively released the Bi-MOF core. This nanosystem achieved potent anti-infective efficacy through three synergistic mechanisms: (1) Bi-MOF exhibited broad-spectrum bactericidal activity against antibiotic-resistant *H. pylori*; (2) modulation of cytokine expression and ROS scavenging alleviated inflammation and oxidative stress; (3) preservation of gut microbiota homeostasis circumvented dysbiosis. In vivo experiments demonstrated a statistically significant inhibition of *H. pylori* colonization without disrupting beneficial gut flora, representing a groundbreaking strategy to combat antibiotic resistance and enhance therapeutic safety [46].

In addition, Wan developed a MOF-based miniature rocket powered by a zinc-powered engine and equipped with a poly (3,4-ethylenedioxythiophene) shell and pH-sensitive enteric-coated coating to achieve targeted therapy. The design autonomously penetrated the gastric mucus layer through the bubble propulsion mechanism while releasing the MOF-loaded drug ingredients, which achieved long-term drug retention for 48 h in the body. This biocompatible design provided an innovative solution for active targeted drug delivery and controlled release in acidic environments and showed a promising application in the biomedical field (Figure 3a) [36].

Combination therapies using nanomedicines demonstrated enhanced therapeutic outcomes. For example, silver nanoparticles (AgNPs) combined with bismuth derivatives, such as colloidal bismuth subcitrate (CBS), and conventional antibiotics showed synergistic effects: AgNPs generated reactive oxygen species (ROSs) to disrupt bacterial membranes, while bismuth compounds inhibited urease activity. This dual approach significantly suppressed *H. pylori* growth, reduced drug resistance, and improved treatment success rates [65].

### 3.4. Biomembrane-Coated Nanoparticles

Additionally, biomimetic membrane-coated nanomaterials have been widely applied in gastritis treatment due to their unique biocompatibility and targeting capabilities. Angsantikul coated PLGA nanomaterials with gastric epithelial cell (such as AGS cells) membrane derivatives and encapsulated antibiotics inside, resulting in AGS-NPs. These nanoparticles carry AGS surface antigens, allowing them to adhere to *H. pylori* in the stomach and achieve targeted drug release. AGS-NPs demonstrably augmented the surface accumulation efficiency of antimicrobial payloads on *Helicobacter pylori* membranes. In vitro assays revealed that fluorescently labeled AGS-NPs exhibited a 10-fold enhancement in specific binding affinity compared to controls, resulting in a significant reduction in *H. pylori* bacterial load. This study demonstrates the feasibility and advantages of using natural host cell membranes to functionalize drug nanocarriers for targeted delivery to pathogens colonizing host cell surfaces (Figure 3d) [66].

### 3.5. Recent Advances in Nanomedicine-Based Therapies for Gastritis

In the past two years, a lot of studies have been conducted on the treatment of gastritis with nanoparticles (Table 1). Sun and colleagues developed a liposome-loaded clarithromycin–bismuth–zinc peroxide hybrid nanoparticle (CLA-Bi-ZnO_2_@Lipo) integrated therapeutic platform targeting multidrug-resistant *H. pylori*. This material achieved rapid drug release triggered by gastric acid, synergistically releasing clarithromycin, Bi^3+^, Zn^2+^, and H_2_O_2_ to exert antibacterial effects. Additionally, it alleviates mucosal inflammation and maintains gut microbiota balance [67]. Additionally, Alam developed a furazolidone-N-acetylcysteine (NAC) liposomal delivery system targeting *H. pylori*. This system significantly improved drug encapsulation efficiency and mucosal penetration, enabling rapid eradication of *H. pylori*. It demonstrated its efficacy in overcoming antibiotic resistance through enhanced drug delivery and synergistic antibacterial effects [68].

Guo developed a self-assembling, pH-responsive hydrogel nanoparticle constructed using the antimicrobial peptide GE33 as its core component, which exhibited stimuli-responsive drug release and bactericidal properties. This material dissociated and released active GE33 monomers in the acidic gastric environment, enabling rapid targeted bacterial killing, while unreleased peptides remained protected by the hydrogel to prolong gastric retention time. This smart delivery system significantly enhanced drug bioavailability, addressing the challenges of short gastric retention time and antibiotic resistance in traditional therapies [69]. Zhou developed a novel gastric acid-responsive antimicrobial hydrogel using acrylic aspartate (AASP) and cysteine-grafted carboxymethyl chitosan (CMCS-NAC) as the matrix, combined with a charge-reversal antimicrobial molecule (C16N-DCA). This hydrogel underwent charge reversal in acidic gastric environments, enabling precise targeting of *H. pylori*. It achieved a 98% eradication rate of *H. pylori* while effectively protecting gastric tissue, modulating the wound microenvironment, and promoting healing [70].

Regarding metallic nanoparticles, a novel pH-responsive zinc-powered MOF microrocket-based drug delivery system was developed, featuring a poly (3,4-ethylenedioxythiophene) (PEDOT) protective layer and enteric coating. This design enabled the system to shield the drug-loaded core in gastric acid environments while autonomously propelling and sustaining drug release in the neutral-pH gastric mucosa. The self-propelled microrockets achieved sustained gastric retention for up to 48 h, prolonging therapeutic drug exposure. This innovation addressed challenges in traditional gastritis treatment, such as poor patient compliance and antibiotic resistance [36]. Yan developed a novel nanomaterial, tannic acid-iron-modified hollow mesoporous silica nanoparticles (THMSNs). This material loaded amoxicillin (Amox) into its iron-containing mesoporous structure. Under gastric acidic conditions, the Fenton reaction was triggered, generating bactericidal hydroxyl radicals for chemodynamic therapy. Simultaneously, the released Amox blocked cell wall repair in damaged bacteria, establishing a synergistic bactericidal mechanism [71].

## 4. Therapeutic Applications of Nanomedicine in Gastric Cancer

### 4.1. Lipid-Based Nanoparticles

Similar to its applications in gastritis treatment, nanomedicine has garnered significant attention in gastric cancer therapy due to its exceptional delivery efficiency. Among various nanocarriers, liposomes have emerged as particularly promising vehicles owing to their superior biocompatibility, biodegradability, and non-immunogenic properties [72]. These vesicular structures not only protect encapsulated drugs from physiological degradation but also prevent off-target effects, thereby minimizing systemic toxicity while enabling efficient delivery of diverse anticancer agents. Berberine (BBR), an isoquinoline alkaloid derived from Coptis chinensis, has demonstrated therapeutic potential against various gastrointestinal disorders, including cancer. However, its intravenous administration is limited by adverse effects such as hypotension, vasodilation, and cardiac depression [73]. To address these limitations, Wang developed PEGylated BBR-loaded liposomes (116.9 nm average diameter) that exhibited enhanced antitumor efficacy (45.8% tumor suppression vs. 38.9% for non-PEGylated formulations) through sustained drug release over 48 h and prolonged circulation time in murine models [74].

Liposomal systems also enable synergistic combination therapy. Ginsenoside Rg3 has been shown to potentiate the anticancer effects of paclitaxel against gastric cancer cells [75]. Building upon this finding, researchers developed an innovative ginsenoside-based liposomal platform where ginsenosides served dual roles as both chemosensitizers and functional membrane components, significantly enhancing tumor growth suppression through coordinated drug delivery. In mouse models, the experimental group exhibited a tumor volume reduction exceeding 80%, approaching nearly complete regression [76].

### 4.2. Polymeric Nanoparticles

**Figure 4:**
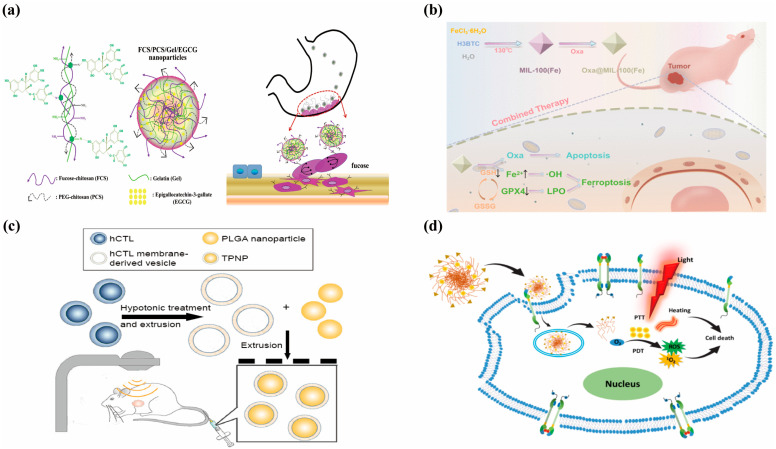
Application of nanomedicine in the treatment of gastric cancer. (**a**) Schematic illustration of the preparation and characterization of FCS/PCS/Gel/EGCG nanoparticles, along with their therapeutic strategy and efficacy evaluation for gastric cancer treatment [reprinted with permission from Ref. [77]; Copyright (2015) Biomacromolecules]. (**b**) Oxaliplatin-loaded MIL-100(Fe) enabling chemotherapy–ferroptosis combined therapy in gastric cancer [reprinted with permission from Ref. [81]; Copyright (2024) American Chemical Society]. (**c**) Schematic of the preparation process of the TPNPs [reprinted with permission from ref. [82]; Copyright (2017) Dove Press]. (**d**) EGFR-targeted nanodrugs for photodynamic therapy (PDT) and photothermal therapy (PTT) in cancer treatment [reprinted with permission from Ref. [84]; Copyright (2021) Elsevier].

Beyond liposomes, chitosan-based biocompatible materials have also been widely applied in the treatment of gastric cancer. Feng’s group engineered an orally administrable nanoplatform for the tumor-targeted delivery of epigallocatechin-3-gallate (EGCG) to gastric carcinoma lesions. The nanocarrier system was constructed through molecular assembly of fucose-functionalized chitosan, PEG-grafted chitosan, and gelatin matrix. In vitro characterization revealed that the sustained EGCG release profile exerted potent antiproliferative effects against malignant cells while triggering programmed cell death pathways. Experimental data demonstrated that the nanoparticles achieved a 54.6% growth inhibition rate against gastric cancer cells in vitro at 40 μg/mL EGCG. In animal models, the tumor volume reduction rate exceeded 80%, and the survival duration of tumor-bearing mice was significantly prolonged (Figure 4a) [77].

### 4.3. Metallic Nanoparticles

In addition, metal–organic frameworks (MOFs) have emerged as versatile drug carriers for gastric cancer treatment due to their exceptional porosity and surface area [78]. Among various MOFs, zeolitic imidazolate framework-8 (ZIF-8) has become particularly prominent owing to its zeolite-like structure, facile synthesis, pH-responsive degradation, and controlled release properties. Wang et al. engineered a hybrid delivery system by encapsulating naringenin (NAR)-loaded ZIF-8 within liposomal bilayers. This NAR@ZIF-8-liposome composite not only prevented premature drug release but also enhanced drug solubility and nanoparticle stability. In vitro experiments demonstrated that the NAR@ZIF-8 liposomes exhibited significantly enhanced inhibition rates against gastric cancer SGC-7901 cells, achieving a drug release efficiency of 79.86% and excellent biocompatibility, thereby providing a promising foundation for clinical translation [79].

Rare earth-based MOFs (RE-MOFs) have attracted growing interest for their combined therapeutic and diagnostic capabilities. Lv et al. developed PEG-modified europium MOFs (DOX@Eu(BTC)) that demonstrated (1) enhanced tumor targeting through prolonged circulation, (2) reduced off-target toxicity, and (3) superior cytotoxicity against multidrug-resistant SGC7901/ADR cells compared to free doxorubicin (DOX). This platform simultaneously inhibited P-glycoprotein expression while inducing apoptosis, offering a promising theranostic approach for resistant gastric cancer [80].

MOFs have also been frequently employed in combination with ferroptosis for gastric cancer treatment. Jiang’s research team developed a nanocomposite termed Oxa@Mil-100(Fe), comprising the chemotherapeutic agent oxaliplatin (Oxa) loaded onto the MOF material Mil-100 (Fe). Mil-100 (Fe) was synthesized via a hydrothermal method, and its porous structure and high specific surface area enabled efficient drug loading, achieving a drug loading capacity of 27.2%. The nanocomposite responded to the acidic conditions and excess glutathione (GSH) in the tumor microenvironment (TME), triggering the release of oxaliplatin and Fe^3+^ ions. Oxaliplatin inhibited DNA synthesis to induce apoptosis in gastric cancer cells, while Fe^3+^ was reduced to Fe^2+^, depleted GSH, inhibited glutathione peroxidase 4 (GPX4) activity, and induced ferroptosis (manifested by elevated lipid peroxides (LPO) and ROS). In vitro experiments demonstrated an 80% inhibition rate against the gastric cancer cell line SGC-7901. In vivo studies using a subcutaneous xenograft tumor model in mice showed that the nanocomposite inhibited tumor growth by nearly 60% through synergistic chemo-ferroptosis mechanisms, with no significant toxicity observed. This strategy enhanced therapeutic efficacy against gastric cancer through synergistic mechanisms and exhibited favorable biosafety (Figure 4b) [81].

### 4.4. Biomembrance-Coated Nanoparticles

Gastric cancer chemotherapy is constrained by drug resistance and systemic toxicity. Zhang developed a biomimetic nanosystem comprising human cytotoxic T-lymphocyte membrane (hCTL)-coated PLGA nanoparticles (TPNPs) to enhance targeted paclitaxel delivery. The hCTL membrane, featuring innate adhesion molecules (LFA-1/CD11a) and immune-evasive properties, replicated native hCTL’s homing capability while reducing macrophage phagocytosis (23.99%) and prolonging circulation. LFA-1-mediated binding to tumor vascular ICAM-1 facilitated nanoparticle accumulation at the tumor site. Combined with localized low-dose radiotherapy (LDI), this strategy synergistically improved paclitaxel efficacy in gastric cancer xenografts, achieving an 88.5% tumor growth inhibition rate and complete remission in two mice. Mechanistic studies revealed LDI-induced ICAM-1 upregulation and vascular normalization, which enhanced nanoparticle penetration. This approach integrates biomimetic targeting, radiotherapy modulation, and controlled drug release, offering a promising solution for precision oncology (Figure 4c) [82].

### 4.5. Recent Advances in Nanomedicine-Based Therapies for Gastric Cancer

Recent studies have extensively explored nanoparticle-based therapies for gastric cancer (Table 2). Zhu developed a novel immunotherapeutic material, LLI liposomes (Liproxstatin-1 and Icy7-loaded liposomes). This system synergistically enhanced ROS levels in the tumor microenvironment (TME) via photodynamic therapy (PDT), inducing immunogenic cell death (ICD) in cancer cells. Simultaneously, Liproxstatin-1 inhibited neutrophil ferroptosis, thereby reversing immunosuppression by blocking T cell exhaustion [83]. Surface engineering of nanocarriers enables active tumor targeting to improve therapeutic precision. Aptamer-functionalized systems like AS1411-conjugated siRNA-loaded lipid nanoparticles (As@LNPs; 183 nm diameter, 85% encapsulation efficiency, 4.6% drug loading) significantly enhanced tumor growth suppression and apoptosis induction in preclinical models through targeted delivery (Figure 4d) [84]. Kim developed a novel TMPRSS4-cleavable peptide (TCP)-based liposome (TCP-L) that utilized the proteolytic activity of tumor-overexpressed transmembrane protease TMPRSS4 for targeted drug delivery. Experimental results demonstrated that DOX-loaded TCP-L significantly enhanced drug release in cancer cells in vitro and showed optimal tumor suppression efficacy in gastric cancer xenograft models [85].

Rouhi developed a polymer-based nanoparticle delivery system targeting the CD44 receptor: Gingerol-HA-PLGA-PEG-NPs (abbreviated as Gingerol-HA-NPs) for gastric cancer therapy. Experimental studies demonstrated that these nanoparticles exhibited significant cytotoxicity against gastric cancer cells, highlighting their potential as a targeted therapeutic platform for gastric cancer [86].

Kong developed a novel targeted nanomedicine delivery system for gastric cancer patients: CPP10-PEG@CUR@FT (CCF). This system incorporated a Fe-TCPP (FT) MOF carrier loaded with the natural anticancer agent curcumin (CUR), modified with CPP10-PEG for active targeting. By integrating chemotherapy (via CUR) and PDT (via FT as a photosensitizer) synergistically, the CCF system significantly enhanced CUR’s bioavailability and tumor-targeting efficiency. Under light irradiation, it exhibited potent cytotoxicity against gastric cancer cells while demonstrating low toxicity, high biosafety, and minimal side effects [87].

Zhang developed a novel biomaterial based on a chitosan/pectin hybrid hydrogel loaded with gold nanoparticles (Au NPs@CS-Pec). This material exhibited significant antiproliferative effects against gastric cancer cell lines and effectively eliminated cancer cells at high doses [88].

Luo developed a P-selectin platelet membrane-coated copper single-atom nanozyme/cisplatin complex (PSC nanoparticles). By integrating the catalytic therapy of single-atom nanozymes (peroxidase, POD activity), photothermal effects, and cisplatin-mediated chemotherapy/NOX-driven H_2_O_2_ generation, this system achieved dual-targeted therapy against primary tumors and metastatic lymph nodes that highly expressed CD44 [89].

Meanwhile, the multifunctional nature of nanomedicines enables their integration with various therapeutic modalities (such as chemotherapy and radiotherapy) to achieve synergistic effects. For instance, certain nanoplatforms can co-deliver chemotherapeutic agents and photosensitizers, significantly enhancing antitumor efficacy through combined PDT [90]. Current research continues to advance nanomedicine development, with promising potential to provide more effective treatment strategies for gastric cancer patients in the near future [91].

## 5. Clinical Trials of Nanomedicines in Gastric Cancer

Despite the promising therapeutic potential of nanomedicines in gastric cancer treatment, significant challenges remain in their clinical translation. Currently, clinical applications of nanomaterials in gastric cancer therapy primarily focus on liposomal formulations and albumin-bound nanoparticle drugs (Table 3).

### 5.1. Liposomal Paclitaxel (L-PTX)

Paclitaxel, a microtubule-targeting agent recommended by NCCN guidelines as a first-line therapy for advanced gastric cancer, has been reformulated as L-paclitaxel using cholesterol/phospholipid membranes to enhance stability and sustained release. Clinical studies demonstrated a 51.43% objective response rate in advanced gastric cancer patients, with primarily grade 1–2 adverse events (hematologic toxicity, nausea, vomiting, and fatigue) and no treatment-related mortality [92]. Compared to conventional paclitaxel, the liposomal formulation reduces solvent-related hypersensitivity and neurotoxicity while maintaining therapeutic efficacy.

### 5.2. Albumin-Bound Nanoparticles

Albumin-bound paclitaxel (nab-paclitaxel) encapsulates paclitaxel molecules within albumin shells, reducing toxicity while improving stability. Its mechanism involves microtubule stabilization to inhibit tumor cell division. Clinically approved for recurrent ovarian cancer and metastatic breast cancer, nab-paclitaxel shows promise in gastric cancer treatment. A study combining nab-paclitaxel with FOLFOX/5-FU reported 16.3% complete response and 38.8% partial response rates with manageable toxicity in gastric adenocarcinoma [93].

Another phase II trial evaluated the efficacy and safety of nab-paclitaxel combined with S-1 as a first-line chemotherapy regimen for gastric cancer [94]. A total of 73 patients were included in the trial. The results showed that an objective response rate ORR) was 58.9%, the median progression-free survival (MPFS) was 9.6 months, and the median overall survival (MOS) was 14.6 months. In terms of safety, 22 patients (30.1%) experienced grade 3/4 toxicity, mainly including neutropenia (12.3%), anemia (5.5%), diarrhea (6.8%), vomiting (2.7%), and peripheral neuropathy (1.4%). No fatal adverse events occurred. This study showed that the nab-paclitaxel combined with the S-1 chemotherapy regimen has good efficacy and controllable safety. It is an effective and safe first-line treatment for patients with metastatic gastric adenocarcinoma.

## 6. Conclusions

As an emerging technological approach, nanomedicine has demonstrated promising potential in the treatment of gastritis and gastric cancer. Leveraging advanced formulation and drug delivery technologies, nanomedicines can significantly enhance therapeutic outcomes. For instance, nanocarriers can improve drug accumulation within tumor tissues while reducing systemic toxicity, thereby enhancing patient quality of life. Furthermore, nanomedicine enables targeted drug delivery, allowing precise action at the disease site while minimizing damage to healthy tissues—an especially critical advantage in the treatment of gastric disorders.

Despite its encouraging performance in theoretical and experimental studies, nanomedicine still faces numerous challenges. Key issues such as the biocompatibility, toxicity, and long-term effects of nanomaterials require further in-depth investigation. Moreover, the translation of nanomedicine from bench to bedside remains a significant hurdle. Effectively integrating nanomedical innovations into clinical practice represents a crucial direction for future research.

To advance the field, future efforts should focus on addressing these challenges, thereby promoting the maturation and broader application of nanomedicine and ultimately benefiting a larger population of patients.

## Figures and Tables

**Table 1:** The latest research on the treatment of gastritis with nanoparticles.

Name	Type	Loaded Drug	Mechanism of Action	Ref.
CLA-Bi-ZnO_2_@Lipo	Lipid-based nanoparticles	Clarithromycin	Antibacterial; alleviate mucosal inflammation and maintain the balance of intestinal flora	[67]
Liposomal drug delivery system	Lipid-based nanoparticles	Furazolidone + N-Acetyl Cysteine	Eradicate *H. pylori* and overcome antibiotic resistance	[68]
GE33 Peptide Hydrogel	Polymeric nanoparticles	GE33 Peptide	Improve drug utilization and overcome antibiotic resistance	[69]
AASP-CMCS-NAC-C16N-DCA Hydrogel	Polymeric nanoparticles	C16N-DCA	Eradicate *H. pylori*, protect gastric tissue, and promote wound healing	[36]
MOF-based Microrockets	Metallic nanoparticles	Rhodamine 6G	Targeted mucosal drug delivery and continuous drug release	[70]
TA-FeHMSN@Amox	Metallic nanoparticles	Amox	Eliminate *H. pylori*, reduce the dosage of antibiotics, and protect the intestinal flora	[71]

**Table 2:** The latest research on the treatment of gastric cancer with nanoparticles.

Name	Type	Loaded Drug	Mechanism of Action	Ref.
LLI	Lipid-based nanoparticles	Liproxstatin-1 + Icy7	Inhibit tumor growth and suppress neutrophil ferroptosis	[83]
As@LNPs	Lipid-based nanoparticles	Bmi-1 siRNA	Inhibit tumor growth	[84]
TCP-L	Lipid-based nanoparticles	DOX	Inhibit tumors and enhance drug release	[85]
Gingerol-HA-NPs	Polymeric nanoparticles	Gingerol	Kill gastric cancer cells	[86]
CPP10-PEG@CUR@FT	Metallic nanoparticles	Curcumin	Tumor-targeted, with low side effects and high biosafety	[87]
Au NPs@CS-Pec	Metallic nanoparticles		Eliminate free radicals and inhibit the proliferation of cancer cells	[88]
PSC Nanoparticles	Biomembrane-coated nanoparticles	Cisplatin + single-atom nanozymes (SAZ)	High lymphatic tropism and dual-targeting capability.	[89]

**Table 3:** Experimental results of clinical treatments with different nanomedicines.

Drug/Carrier	Experimental Phase	Treatment Plan	ORR	MOS (Month)	Adverse Reactions
Liposomal paclitaxel		Liposomal Paclitaxel + Cisplatin	51.43%	15.3	Hematologic toxicity, leukopenia (Grade III–IV), gastrointestinal reactions, alopecia (mostly Grade I–II)
Albumin-bound Paclitaxel	Phase II (2018)	Albumin-bound Paclitaxel + FOLFOX			Non-febrile neutropenia (20.4%), nausea (8.2%), diarrhea (8.2%), neuropathy (6.1%)
Albumin-bound Paclitaxel	Phase II (2018)	Albumin-bound Paclitaxel + S-1	58.9%	14.6	Neutropenia (12.3%), anemia (5.5%), diarrhea (6.8%), vomiting (2.7%), peripheral neuropathy (1.4%)
